# Regulation of Cancer Metabolism by Deubiquitinating Enzymes: The Warburg Effect

**DOI:** 10.3390/ijms22126173

**Published:** 2021-06-08

**Authors:** So-Hee Kim, Kwang-Hyun Baek

**Affiliations:** Department of Biomedical Science, CHA University, Seongnam-si 13488, Gyeonggi-do, Korea; kshksh5981@naver.com

**Keywords:** anaerobic glycolysis, anticancer, hypoxia, small molecules, ubiquitin–proteasome system (UPS)

## Abstract

Cancer is a disorder of cell growth and proliferation, characterized by different metabolic pathways within normal cells. The Warburg effect is a major metabolic process in cancer cells that affects the cellular responses, such as proliferation and apoptosis. Various signaling factors down/upregulate factors of the glycolysis pathway in cancer cells, and these signaling factors are ubiquitinated/deubiquitinated via the ubiquitin–proteasome system (UPS). Depending on the target protein, DUBs act as both an oncoprotein and a tumor suppressor. Since the degradation of tumor suppressors and stabilization of oncoproteins by either negative regulation by E3 ligases or positive regulation of DUBs, respectively, promote tumorigenesis, it is necessary to suppress these DUBs by applying appropriate inhibitors or small molecules. Therefore, we propose that the DUBs and their inhibitors related to the Warburg effect are potential anticancer targets.

## 1. Introduction

**Figure 1:**
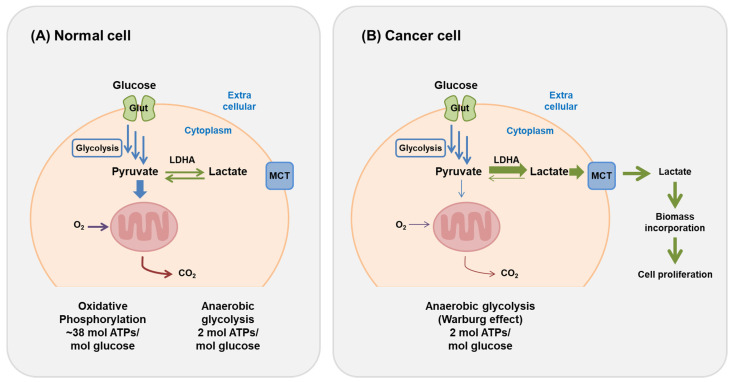
Differences in glycolysis pathways between normal cells and cancer cells. (**A**) In the presence of oxygen, normal cells produce carbon dioxide up to 38 ATPs per glucose molecule through glycolysis, TCA cycle, and electron transport system. In a hypoxic environment, pyruvates are accumulated without going through the TCA cycle. These accumulated pyruvates in the muscle tissue are converted to lactic acid and only produce 2 ATPs. (**B**) Cancer cells only use the glycolysis process, regardless of the presence or absence of oxygen; 2 ATPs are produced per glucose molecule and, therefore, compared to normal cells, more glucose is required to obtain energy.

Cellular respiration is the process by which living organisms decompose organic matter to inorganic matter for producing energy required for survival. Sequentially, this encompasses the glycolysis process, the tricarboxylic acid cycle (TCA cycle, also called Krebs cycle), and the electron transport system [1]. In normal cells, glucose is converted to pyruvate through glycolysis in the cytoplasm, and the pyruvate enters the mitochondria where it is completely degraded [2]. This process requires oxygen, and 38 ATPs are finally produced from one molecule of glucose: 2 ATPs during glycolysis, 2 ATPs in the TCA cycle, and 34 ATPs in the electron transport system [3]. Under hypoxic conditions, the pyruvate is converted to lactic acid, which accumulates instead of entering the TCA cycle. In cancer cells, there is an abnormal progression of metabolism that only utilizes glycolysis. In normal cells, 38 ATPs are generated with one glucose molecule, whereas only 2 ATPs are generated in cancer cells (Figure 1) [4]. Consequently, the cancer cells require more glucose molecules than normal cells to obtain enough energy to survive. This is a remarkable characteristic of cancer cells and has recently been applied as a method of detecting cancer by exploiting the characteristic of excessive glucose utilization by cancer cells [5]. It is essential to consider that cancer cells complete the glycolysis process regardless of absence or presence of oxygen [6]. When cancer cells only obtain glycolysis-dependent energy even in the presence of oxygen, the effect is called the “Warburg effect” [7].

Homeostasis, which is a property of maintaining a constant state in response to various stimuli in an individual or a cell, is an essential factor. However, the imbalance of homeostasis leads to various diseases, including cancer. Therefore, it is important to maintain an optimized state by restoring an equilibrium state broken by changes in the surrounding environment. The degradation and synthesis of proteins in cells are an example of maintaining homeostasis. Proteins that need to be discarded after their half-life, and unstable proteins due to damage, are degraded. Here, ubiquitin serves a marker for labeling proteins that need to be degraded. Ubiquitin is finally covalently bound to the target protein through a series of processes using the E1, E2, and E3 enzymes [8]. Ubiquitin molecules attached to the substrate form a polyubiquitin chain, regulate the activity and function of the substrate protein, and induce degradation through the 26S proteasome [9]. The E3 ligase plays a role in attaching ubiquitin to the target protein, whereas the deubiquitinating enzyme (DUB) induces a reversible reaction that breaks the bond between the target protein and ubiquitin or between the ubiquitin [10]. The ubiquitinated proteins mediated by an E3 ligase are degraded by the 26S proteasome, thereby reducing the cellular functions of the proteins. Alternatively, DUBs stabilize the substrate proteins and improve their cellular functions by modulating the degradation [11]. Thus, degradation and expression of proteins by the ubiquitin–proteasome system (UPS) play an important role in cell homeostasis [12] and can be used as an anticancer drug to remove oncoproteins or stabilize tumor suppressor proteins through UPS [13].

## 2. Cancer Metabolism Involved in the Warburg Effect

### 2.1. Changes in the Tumor Microenvironment

Unlike normal cells, cancer cells require considerable energy to replicate due to their inherent characteristic of abnormally rapid proliferation [14]. The tumor microenvironment (TME), such as hypoxia, results in metabolic changes, including the Warburg effect, in cancer cells. Alterations of the tumor microenvironment due to HIF initiate transcription programs under hypoxic stress conditions [15]. HIF is a transcription factor that regulates angiogenesis, and heterodimers include HIF-1, HIF-2, and HIF-3. HIF-1α is expressed in all cells, whereas HIF-2α is expressed in endothelial cells or hepatocytes and controls angiogenesis and red blood cell production [16]. The level of hypoxia-induced neovascularization varies and is dependent on the expression levels of ANGPT-2 and VEGF-A [17]. In non-small-cell lung cancer cells, inhibition of the HIF-1α/VEGF signaling pathway by GLA inhibits the hypoxia-induced cell invasion and proliferation [18]. The representative target of pVHL is HIF. pVHL binds to elongin C and CUL2, thus forming the VCB–CUL2 complex with elongin B. Under normal oxygen conditions, acetylation and hydroxylation of proline residues in the oxygen-dependent degradation domain (ODD) of HIF-α induces binding to the VCB–CUL2 complex and functions as an anti-tumor when HIF-1α is degraded via the ubiquitin–proteasome pathway [19]. Under hypoxia, the HIF-1α is not degraded, accumulates in the cells, and subsequently interacts with HIF-β. Blood supply to all cancer cells is restricted with tumor progression, and the tumor microenvironment stabilizes HIF-1α and HIF-2α [20,21], which are therefore found to be upregulated in tumors; however, this does not apply to all cancer cell types. In lung adenocarcinoma, HIF-1α is upregulated, but HIF-2α is downregulated [22].

### 2.2. Changes in the Signaling Pathways

AKT is an important signaling molecule that induces the Warburg effect in cancer cells. PI3K phosphorylates and activates AKT located on the cell membrane [23]. The phosphorylated AKT separates from the cell membrane, causing intracellular reactions with the mTORC-containing substrates. AKT promotes lipid biosynthesis and glucose transport to cancer cells with improved glycolysis through activation of mTOR [24]. The PI3K/AKT signaling pathway promotes the breakdown and uptake of glucose and lactic acid production, thus playing an important role in the metabolic reprogramming of cancer cells [25]. It also regulates the growth, proliferation, and apoptosis of cancer cells by stabilizing, inhibiting, and regulating downstream factors [26]. Growth factors regulating HIF-1α activate the PI3K/AKT signaling pathway [27]. Conversely, hindrance of the AKT signaling pathway inhibits glycolysis and prevents cell growth. CTMP inhibits AKT phosphorylation with subsequent inhibition of the PI3K/AKT pathway [28], and FDFT1 negatively regulates the AKT/mTOR signal to inhibit glycolysis and proliferation in colon cancer [29]. Moreover, PTEN also acts as a negative regulator of the PI3K/AKT pathway [30].

### 2.3. Change from Oxidative Metabolism to Reduced Metabolism

NADH is an important factor involved in altering the redox metabolism of cells and is used by the mitochondria for electron transport. NAD^+^ is regenerated from NADH by a redox-linked mitochondrial shuttle, of which malate-aspartate is the most commonly recognized shuttle [31]. During glycolysis, a malate-aspartate shuttle through the mitochondria restores the NADH imbalance. However, when transport through the intracellular membrane attains the maximum rate, the shuttle exceeds the range that accommodates glycolysis and subsequently converts pyruvate to lactic acid via LDH to produce NAD^+^ [32]. The conversion of NADH to NAD+ in the cytoplasm satisfies the redox imbalance occurring in the glycolysis process, and the mitochondrial NADH produced enters the electron transport system and eventually produces more ATPs [33]. Thus, the balance of NADH redox contributes to a direct signaling role for the Warburg effect [34]. Inhibition of lactate dehydrogenase A (LDH-A) suppresses the Warburg effect and returns to oxidative phosphorylation to re-oxidize NADH and produce ATP [35].

### 2.4. Consumption of Glutamine

The Warburg effect is a major metabolic characteristic of cancer. Proliferating cells require not only ATP but also other cellular components. Along with glucose, glutamine is one of the most abundant nutrients in plasma. Similar to glucose, glutamine is degraded to lactic acid and does not undergo complete oxidative phosphorylation in mammalian cells. Glutamine is also utilized in biosynthetic pathways that provide a secondary carbon source for fatty acid synthesis. Glutamine-derived α-ketoglutarate is supplied for the production of citrate through forward flux via the TCA cycle and malic enzyme-dependent production of pyruvate [36].

### 2.5. Lipid Biosynthesis

Lipids are partially derived from acetyl CoA and many contain fatty acids (FAs), which are essential for cancer cell proliferation. Adipocytes provide FAs for rapid tumor growth. FAs are considered a major source of electrons for ATP production and are used to produce energy in cancer cells [37]. In addition, mitochondrial fatty acid oxidation (β-oxidation) produces flavin adenine dinucleotide (FADH2) and nicotinamide adenine dinucleotide (NADH) via oxidative phosphorylation (OxPhos). A recent study found that a lack of glucose in cancer cells does not reduce ATP levels; however, blocking fatty acid oxidation (FAO) showed no effect in normal cells but reduced ATP production in cancer cells by 40% [38]. Further studies are required to determine which pathways among glucose, glutamine, and fatty acid metabolism are primarily used by cancer cells to produce ATP. In addition, cancer cells are known to improve biosynthetic ability by expressing pyruvate kinase isozymes M2 (PKM2) [39].

### 2.6. Drug Resistance

The inflow of glucose into cells is promoted by 14 GLUT transporter groups. GLUT1 and GLUT3 have been extensively investigated for cancer metabolism [40,41]. GLUT1 is upregulated in malignant tumors, such as prostate cancer [40] and breast cancer [42]. Increased GLUT1 expression activates mTOR [43], and activated mTOR increases the expression of GLUT1 [44], thereby forming a positive feedback loop. LoVo cells resistant to doxorubicin (an anticancer drug) showed a higher dependence on glucose metabolism and expression of GLUT1 and MCT4 for survival as compared to doxorubicin-sensitive LoVo cells. Silybin, an inhibitor of GLUT, is reported to decrease the expression of the GLUT protein. Exposure to 50 μM silybin resulted in decreased expression of GLUT1 only in the doxorubicin-resistant cells; however, exposure to 10 μM silybin affected both doxorubicin-sensitive and -resistant cells [45]. This result indicates that upregulation of GLUT is a target of resistance to drugs, such as doxorubicin. GLUT3 is highly expressed in brain tumor cells [46], and long-term treatment with temozolomide in human astrocytes resulted in increased expression of GLUT3, indicating that GLUT3 has acquired resistance to the temozolomide drug [47].

## 3. Ubiquitin–Proteasome System and DUBs

**Figure 2:**
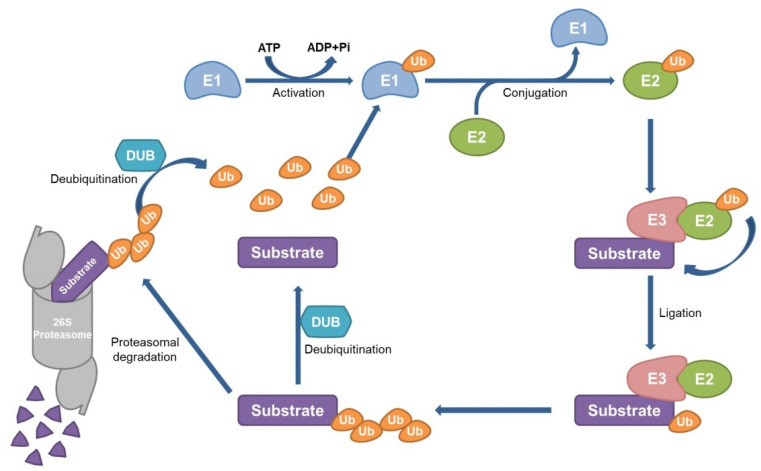
The schematic diagram of the ubiquitin–proteasome system (UPS). The ATP-activated E1 enzyme binds to glycine at the end of ubiquitin. The E1 enzyme delivers the ubiquitin to the E2 enzyme. The E2 enzyme binds to the E3 enzyme (E3 ligase) bound to the substrate protein. Ubiquitin linked to the E2 enzyme moves to the substrate protein. By repeating this process, several ubiquitins form a polyubiquitin chain, and the substrate is degraded through the 26S proteasome. Deubiquitinating enzyme (DUB) acts in opposition to the E3 ligase, which links ubiquitin to the substrate protein.

Ubiquitin is a small protein expressed in all eukaryotes, is composed of 76 amino acids, and is covalently bound to substrates. Ubiquitination is a post-translational modification that has various roles in cellular processes, including cell cycle, DNA repair, and signal transduction [48]. Ubiquitination is a multistep enzyme cascade that functions via the E1 (ubiquitin-activating enzyme), E2 (ubiquitin-conjugating enzyme), and E3 ligase (ubiquitin ligase) [49]. The glycine at the ubiquitin end first links to the cysteine residue of the E1 enzyme. ATP is required for this process, and the activated E1 enzyme transfers the linked ubiquitin to the E2 enzyme, which then binds to the E3 enzyme and transmits ubiquitin to the target protein [50]. The polyubiquitin chain formed by repeating this process leads the target protein to the 26S proteasome and eventual degradation of substrates [51]. This process is defined as the ubiquitin–proteasome system (UPS). The E3 enzyme is also called the E3 ligase, which links the ubiquitin to a target protein in UPS; other enzymes that function opposite to E3 ligase are called the deubiquitinating enzymes (DUBs) (Figure 2) [12]. Ubiquitin has the capability to form a polyubiquitin chain at 7 Lys sites and 1 Met site: K6, K11, K27, K29, K33, K48, K63, and M1. It is well known that the K6-linked polyubiquitin responds to mitophagy and DNA repair [52]. K11-linked polyubiquitin is known to control the cell cycle, proteasomal degradation, protein stability, mitophagy, trafficking, and endoplasmic reticulum-associated protein degradation [52,53,54]. K27-linked polyubiquitin activates kinases and regulates DNA repair [54]; K29-linked polyubiquitin plays a role in kinase modification and proteasomal/lysosomal degradation [55]; and K33-linked polyubiquitin induces kinases modification, innate immunity, and autophagy [54,56]. It is also known that K48-linked polyubiquitin induces proteasomal degradation [57], and the K63-linked polyubiquitin plays a role in protein kinase activation and DNA damage [58]. Lastly, M1-linked polyubiquitin activates gene expression and innate immunity [52].

DUBs are involved in two major roles. The first is to break the bond between ubiquitin and a protein, so that the protein from which ubiquitin has been removed can continue to function in the cells. The second involves breaking the bond between ubiquitin and ubiquitin [59]. After the target protein enters the proteasome, the remaining ubiquitins are broken down one by one in the form of a chain, and free ubiquitin is recycled back to the UPS. DUBs are classified into nine subfamilies [60]: ubiquitin-specific protease (USP), ubiquitin C-terminal hydrolases protease (UCH), Machado–Joseph disease protein domain protease (MJD), ovarian tumor protease (OTU), Jab1/Pab1/MPN metallo-enzyme motif protease (JAMM), monocyte chemotactic protein-induced protease (MCPIP), permuted papain fold peptidase of dsDNA viruses and eukaryotes (PPPDE), motif interacting with Ub-containing novel DUB family (MINDY), and zinc finger with UFM1-specific peptidase domain protein (ZUFSP, also called ZUP1 or C60rf113) [61,62,63]. The USP, UCH, OTU, MJD, MCPIP, PPPDE, MINDY, and ZUFSP subfamilies have cysteine peptidase activity, whereas the JAMM subfamily contains zinc metalloisopeptidase activity [64,65].

## 4. DUBs of the Warburg Effect Factors

### 4.1. USP7

Ubiquitin-specific protease 7 (USP7), also called the herpesvirus-associated ubiquitin-specific protease (HAUSP), is a protein belonging to USP, the largest subfamily of DUB. It targets proteins associated with numerous tumors, such as p53 [66], MDM2 [67], PTEN [68], FOXO4 [69], and histone H2B [70], and is responsible for various biological functions, such as tumor suppression, DNA repair, immune response, and apoptosis [71].

SIRT7, a new target of USP7, plays a role in controlling a variety of cellular processes, ranging from cell homeostasis [72], senescence [73], and DNA repair to cancer progression [74]. It also acts to resist various types of stress, such as hypoxia [75], endoplasmic reticulum stress [76], and low glucose levels [77]. USP7 is a DUB of SIRT7 and decreases the K-63 linked ubiquitination level of SIRT7 in HCT116 cells. However, the binding affinity of USP7 and SIRT7 is found to decrease in glucose starvation, and the ubiquitin level of SIRT7 decreases with increasing glucose concentration. SIRT7 deubiquitination by USP7 inhibits the expression of G6PC, a key regulator of SIRT7-mediated glucose production [78].

FoxO1, a member of the FoxO subfamily of forkhead/winged helix transcription factors, is important for inhibition of the insulin-mediated glucose production [79]. FoxO1 interacts with the insulin responsive enzyme (IRE) in the promoter regions of G6PC and Pck1 [80]. Insulin signaling inhibits the transcriptional activity of FoxO1 through AKT-dependent phosphorylation of FoxO1 at certain conserved residues (T24, S256, and S319 of human FoxO1) [81]. USP7 deubiquitinates the FoxO1 and inhibits transcriptional activity in HEK293A cells. In addition, USP7 regulates FoxO1 occupancy in the promoter of the glucose-generating gene [82]. Thus, the activity of USP7 alleviates excessive glucose production in a hypoxic environment.

### 4.2. USP19

The ubiquitin-specific protease 19 (USP19) protein belongs to the USP subfamily. USP19 promotes tumor formation in gastric cancer by upregulating MMP2 and MMP9 involved in cancer migration and invasion [83] and regulates the growth of Ewing sarcoma by acting as a DUB of the chimeric transcription factor EWS-FLI1 [84]. USP19 not only regulates cancer cells but also performs various functions within cells, such as differentiation of muscle cells [85], viral immune response [86], autophagy [87], macrophage polarization [88], cell cycle regulation [89], chromosome stabilization, and repair of DNA damage [90].

HIF is a transcription activator having subunits HIF-α (HIF-1α, HIF-2α, and HIF-3α) and HIF-β, and mediates the response of cell proliferation/survival, angiogenesis, and glucose metabolism [91]. USP19 interacts with HIF-1α and stabilizes HIF-1α, regardless of the ER localization and catalytic activity of USP19 in HeLa cells. Knockdown of USP19 decreases the protein levels of HIF-1α and the mRNA expression levels of *VEGF* and *GLUT1*, which are target genes of HIF-1α in hypoxia [92].

### 4.3. USP28

The ubiquitin-specific protease 28 (USP28) protein belongs to the USP subfamily and USP28 responds to DNA damage and stabilizes proteins such as Myc and cyclin E [93]. In addition, USP28 is overexpressed in tumors of human colon cancer patients and acts as an oncogene that promotes the formation of intestinal tumors [94].

Fbw7, a substrate of USP28, is an E3 ligase that plays a role in Myc- or cyclin E-mediated cancer development [95]. Loss of Fbw7 in mice decelerates the cardiovascular development and increases embryonic mortality via an increase in the *notch1* mRNA levels [96]. USP28 does not directly bind to c-Myc but deubiquitinates the c-Myc through interaction with Fbw7 in vivo and in vitro, the E3 ligase of c-Myc [97].

### 4.4. USP37

The ubiquitin-specific protease 37 (USP37) protein belongs to the USP subfamily. USP37 has a role in regulating the cell cycle and accelerates conversion of the G1/S phase. USP37 is also reported to be a transcriptional target of the oncogenic transcription factor E2F1, which is upregulated in several cancers [98]. Recent studies report that USP37 is a potential factor related to breast cancer progression [99].

The *Myc* oncogene, which contributes to the development of numerous human cancers, encodes the transcription factor c-Myc, thereby linking altered cellular metabolism with oncogenesis [100]. c-Myc plays an important role in promoting glycolysis in the Warburg effect [101] and cooperates with E2F1 in regulating the expression of genes involved in nucleotide metabolism. Along with HIF-1, it is reported to regulate the expression of genes involved in glucose metabolism [102,103]. The overexpression of c-Myc can be controlled by the UPS. c-Myc is deubiquitinated and stabilized by direct binding to USP37 in HEK293T cells. Moreover, depletion of USP37 results in decreased expressions of GLUT1 and LDHA mRNAs required for glucose uptake and lactic acid production in H1299 cells. This suggests that USP37 regulates the Warburg effect via stabilizing c-Myc in lung cancer [104]. Interestingly, unlike USP28 [97], USP37 directly binds to c-Myc and acts independently of Fbw37.

### 4.5. USP44

The ubiquitin-specific protease 44 (USP44) belongs to the USP family and is a DUB containing a zinc-finger domain and a USP domain. USP44 is involved in numerous cellular functions, including stem cell differentiation [105] and central body position regulation [106], and responses to DNA damage [107]. In addition, USP44 plays an important role in human tumors acting as both a tumor suppressor and an oncogene. It inhibits cell growth by inhibiting AKT signaling in non-small-cell lung cancer [108] and promotes the growth of prostate cancer by stabilizing the EZH2 protein [109].

Fructose-1, 6-bisphosphatase 1 (FBP1) is an enzyme that regulates glucose production and catalyzes the decomposition of fructose 1, 6-bisphosphate into fructose 6-phosphate and inorganic phosphate. FBP1 acts as a tumor suppressor in diverse malignancies, such as kidney cancer [110], breast cancer [111], lung cancer [112], and liver cancer [113]. Knockdown of USP44 improves glucose utilization and lactic acid production capacity by reducing only FBP1, and not HK2 or PKM2, in PANC-1 cells. Conversely, overexpression of USP44 increases the protein expression level of FBP1. This suggests that FBP1 mediates USP44 and plays an important role in glucose metabolism [114].

### 4.6. OTUB2

Ubiquitin aldehyde binding 2 (OTUB2) belongs to the OTU subfamily, which relies on DNA damage to fine-tune the ubiquitination level and supports the DNA repair pathway selection [115]. In addition, OTUB2 promotes cancer metastasis by activating YAP and TAZ, independent of the Hippo signaling pathway [116].

AKT, also known as protein kinase B (PKB), is a serine/threonine kinase that induces cell growth and differentiation. AKT is capable of phosphorylating various downstream factors related to apoptosis, transcription, and oncogene [117]. Phosphorylation-induced AKT activation, overexpression, and mutation are frequently observed in human cancers [118]. Knockdown of OTUB2 reduces glycolysis and its ability to absorb glucose or produce extracellular lactic acid in A549 and H1299 cells. In NSCLC cells, the expressions of HIF-1α and c-Myc are decreased. In addition, OTUB2 increases the phosphorylation levels of mTOR and AKT and protein expression levels of GLUT1, U2AF2, HK2, PGAM1, PGK1, HIF-1α, and c-Myc in XL-2 cells. This indicates that the mTOR/AKT signaling pathway can be activated by OTUB2 and suggests that OTUB2 is a regulator of the Warburg effect through interaction with U2AF2 [119].

### 4.7. OTUD6B

A double allele mutation in the *ovarian tumor domain-containing 6B (OTUD6B)* gene is associated with development of the intellectual disability syndrome [120]. Additionally, the long noncoding RNA OTUD6B-AS1 (lncRNA OTUD6B-AS1) of OTUD6B exacerbates oxidative damage in bladder cancer [121] and inhibits the growth, invasion, and migration of thyroid cancer [122]. The lncRNA OTUD6B-AS1 also regulates the Wnt/β-catenin signaling pathway to induce the proliferation and invasion of hepatocellular carcinoma [123]. However, it contrarily inhibits the proliferation of renal cell carcinoma [124].

The protein von Hippel–Lindau (pVHL) is an E3 ligase that plays a role as a tumor suppressor. *pVHL* is present on chromosome 3(3p25–26) and mutations cause von Hippel–Lindau disease, a genetic disease that is transmitted through generations [125]. In HCC cells with OTUD6B knockdown, the protein levels of HIF-1α and HIF-2α were increased in both the normal oxygen state as well as the hypoxic state. In addition, overexpression of OTUD6B resulted in increased ubiquitination of HIF-1α in MHCC-LM3 cells. However, OTUD6B did not bind to HIF-1α but instead directly bound to pVHL. OTUD6B binds to elongin B and enhances the interaction with pVHL-elongin C, thereby inhibiting pVHL from proteasome degradation in HEK293T cells. The stabilized pVHL recognizes and degrades hydroxylated or SUMOylated HIF-1α in hypoxia. As a result, OTUD6B increases the stability of pVHL to prevent metastasis of hepatocellular carcinoma [126,127].

### 4.8. OTUD7B

The OTU domain-containing protein 7B (OTUD7B), also called Cezanne, is a member of the OTU subfamily that contains the OTU domain. OTUD7B positively modulates and activates T cells and induces an inflammatory response [128]. Moreover, by inhibiting the NF-κB signaling pathway, OTUD7B negatively regulates and inhibits B cell activity [129]. OTUD7B not only responds to the immune system but also functions as an oncogene; OTUD7B induces lung squamous carcinoma via the AKT/VEGF signaling pathway [130] and activates the NF-κB signaling pathway to increase resistance to apoptosis in hepatocellular carcinoma [131]. 

**Figure 3:**
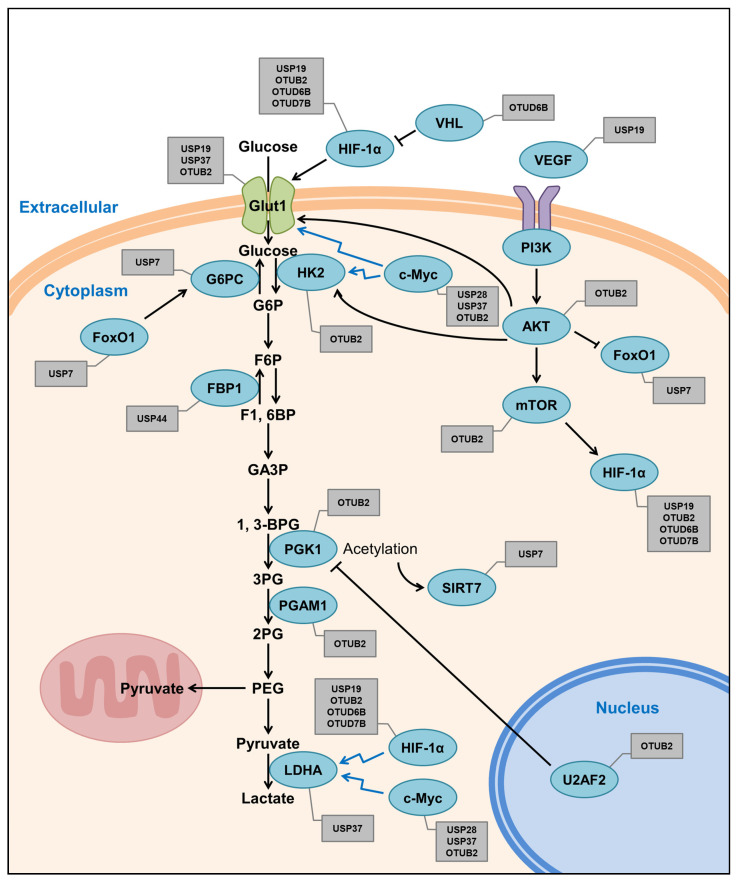
The schematic model for the Warburg effect modulated by DUBs and their substrates. The black arrow indicates direct stimulatory/inhibitory modification, and the blue arrow indicates transcriptional stimulatory modification. The blue circles represent the factors of the Warburg effect, and the gray square boxes represent their DUBs.

Under hypoxia, HIF-1 upregulates the expression of Pdk1, an enzyme regulating glycolysis in cancer cells and *VHL*-deficient osteoblasts, and promotes the conversion of cytoplasmic pyruvate to lactic acid [132]. Knockdown of OTUD7B decreases the protein levels of HIF-1α in HeLa cells and mouse embryonic fibroblasts (MEFs) isolated from OTUD7B knockout mice (OTUD7B−/−) and increases the K11-linked polyubiquitin chain formed. However, it is confirmed that HIF-1α, which is degraded by OTUD7B, is regulated through a proteasome-independent process in OTUD7B knockdown cells (Table 1 and Figure 3) [133]. 

### 4.9. Other DUBs

There are DUBs excluded from this paper because they are not identified to be related to the Warburg effect, glycolysis, or cancer, yet. For example, USP1 limits PI3K–AKT–FoxO signaling by removing the K63-linked polyubiquitin chain from AKT in prolonged starvation mouse muscle and depletion of USP1 increases ubiquitination level of AKT, glucose uptake, and PI3K–AKT–FoxO signaling [134]. However, different results may occur when applied to humans or other diseases such as cancer, because DUBs function differently depending on the cell types. Another DUB that regulates AKT, UCH-L1, induces lymphoma by deregulating AKT signaling [135]. However, the effect of UCH-L1 on the Warburg effect or glycolysis is not known yet.

There are other DUBs excluded from this paper for the same reason as described. DUBs which affect c-Myc are CYLD [136], USP2a [137,138,139], USP7 [140,141], USP13 [142], USP16 [143], USP18 [144], USP22 [145,146], USP28 [147], USP36 [148,149], and OTUD6B isoform 2 [150]. DUBs that regulate FBP1 include USP22 [151] and USP44 [114]. The DUBs of HIF-1α are MCPIP1 [152], UCH-L1 [153,154,155], USP7 [156], USP8 [157], USP20 [158], and USP28 [158]. USP4 [159], USP5 [160], USP9X [161], USP10 [162], and OTUD5 [163] regulate mTOR signaling, and USP22 [164] and OTUB1 [165] regulate activity of mTOR.

These DUBs affect signaling pathways, but it is not known how they affect survival, apoptosis, the Warburg effect, or glycolysis in cancer cells. Therefore, follow-up studies related to metabolism in cancer cells and clinical samples are required. 

## 5. Small Molecules of DUBs Associated with the Warburg Effect 

Depending on the target protein, DUBs can play a role in tumor formation as well as suppression. For example, when a DUB acts on the tumor suppressor protein p53, the degradation of p53 is inhibited, and the tumor suppressor role is restored by stabilizing p53 [166,167]. However, if the DUB targets an oncoprotein such as c-Myc, it plays a role in tumorigenesis [97,104,119]. Therefore, inhibiting a DUB, which acts as an oncoprotein, may be another way to inhibit the Warburg effect and treat cancer. Table 2 reveals the inhibitors and small molecules of DUBs featured in this paper (Table 2). However, since inhibitors or small molecules of DUBs are yet to be developed, such DUBs are other potential targets for anticancer therapeutics. 

### 5.1. USP7

FT671 and FT827 are small molecule inhibitors that specifically target USP7 and no other DUBs. FT671 is a non-covalent inhibitor, whereas FT827 is a covalent inhibitor, which binds and inhibits the catalytic domain of USP7. By inhibiting USP7, FT671 induces increased protein levels of p53 and p53 target proteins in HCT116 and U2OS cells and stabilization of the p53 protein and degradation of N-Myc in the IMR-32 cells [168].

GNE-6640 and GNE-6776 non-covalently target 12Å remote from the catalytic cysteine and not the catalytic domain of USP7. GNE-6640 and GNE-6776 interact with the side chain and acidic residues of ubiquitin K48. This means that USP7 binds and decomposes ubiquitin moieties having the side chain of K48 and, consequently, inhibits the activity of USP7 by weakening the binding with ubiquitin [169].

HBX 19,818, HBX 28,258, and HBX 41,108 are small molecule substances that inhibit the activity of USP7, where HBX 19,818 and HBX 28,258 inhibit HAUbVS binding to USP7. In particular, HBX 19,818 covalently binds to the catalytic cysteine C223 of USP7 and increases the deubiquitination level of the USP7-mediated MDM2 and functional activation of p53 [170]. In addition to the p53-dependent apoptosis, HBX 41,108 induces USP7-mediated p53 stabilization and activation. Unlike HBX 41,108, which non-specifically inhibits DUB, HBX 19,818 and HBX 28,258 target USP7 as specific targets [170,171].

P22077 is an inhibitor that jointly targets USP7, USP47, and USP10, and P50429 inhibits USP7 and USP47. P22077 and P50429 covalently modify the catalytic cysteine (C233) of USP7 and promote structural changes of enzymes related to rearrangement of the active site. In addition, P22077 causes a partial loss, but P50429 completely abolishes the link between Ub-vinyl methyl ester (Ub-VME) and USP7, which is an ubiquitin variant that binds to the catalytic cysteine of DUB [173]. The inhibition of USP7 by P22077 results in accumulation of the 26S proteasome complex and polyubiquitinated substances but does not directly block the proteasome proteolytic activity. In the early stage of P22077 treatment, there is a decrease in the USP7-mediated HDM2, with increased protein expressions of p53 and p21; however, in the later stages, there is a decrease in the protein expression level of HDM2 due to the p53/HDM2 feedback loop. In addition, P22077 decreases the expression levels of claspin and Chk1, resulting in a checkpoint arrest [172].

P5091 is a trisubstituted thiophene with dichlorophenylthio, nitro, and acetyl substituents and reacts specifically with USP7, but not with other DUBs. P5091 inhibits USP7 by binding to USP7 competitively with Ub-VME, and inhibiting the deubiquitinating activity of USP7, but not the proteasome [174]. In multiple myeloma (MM) cells treated with P5091, the level of ubiquitination of HDM2 and protein expression levels of HDM2 and HDMX are increased, and the protein levels of p53 and p21 are downregulated. Moreover, there is also a reduction in the angiogenic activity [175].

vif1 and vif2 are peptides derived from Kaposi’s sarcoma-associated herpesvirus (KSHV) vIRF4; vif1 binds competitively with the TRAF domain of USP7, which is known to bind to a substrate having P/A/E-x-x-S, whereas vif2 inhibits the deubiquitination activity of USP7 by binding both the TRAF domain and the catalytic domain of USP7. Treatment with vif1 and vif2 promotes anti-tumor effects via p53-mediated apoptosis and p21-mediated cell cycle arrest [176].

XL177A is an irreversible inhibitor that acts on the catalytic cysteine C223 of USP7. When treated with XL177A, the MCF7 cells expressing USP7 show reduced expression of HDM2 and upregulation in the expression levels of p53 and p21, CDKN1A and GADD45A (proteins that arrest the cell cycle), and Bax and DDB2 (apoptotic proteins) [177]. XL188 is a small molecule inhibitor, which was developed before XL177A, and is reported to inhibit noncovalent active sites. XL188 treatment induces downregulation of HDM2 and upregulation of the p53 and p21 proteins [178].

### 5.2. USP28

AZ1 is a double inhibitor targeting both USP25 and USP28. The fluorophenyl ring of AZ1 forms an arene–H interaction with Phe370, while the bromophenyl ring forms a π–π interaction with Phe370. The fluoro and OH groups form hydrogen bond interactions with the Glu366 and Lys381 residues. Treatment of AZ1 induces apoptosis by downregulating the expression level of the tumor protein c-Myc in cancer cell lines [179]. Binding of the [1,2,3]triazolo[4,5-d]pyrimidine to the catalytic domain of USP28 and attachment of the benzyl group to the triazole ring of [1,2,3]triazolo[4,5-d]pyrimidine are important for activity. [1,2,3]triazolo[4,5-d]pyrimidine inhibits the survival, proliferation, cell cycle, and migration of cancer cells and induces apoptosis through the degradation of c-Myc by the proteasome [180].

### 5.3. Broad Spectrum of DUB Inhibitors

Betulinic acid is a natural anticancer drug derived from birch, which induces apoptosis by inhibiting the proteasome reaction in various cancer cells. Betulinic acid induces apoptosis by activating NF-κB, a major mediator of the cellular stress response [181]. It also upregulates the Bax protein [182], downregulates the expression of VEGF [183], and induces cell cycle arrest [184]. Interestingly, normal tissues and cells are not affected by betulinic acid [185].

PR-619 targets a wide range of DUBs but not all cysteine proteases. PR-619 induces G2/M cell cycle arrest in esophageal squamous cell carcinomas cells (ESCCs) and inhibits cell growth by inhibiting cyclin B1 and stabilizing p21. It also induces ER stress and promotes apoptosis [186]. In fibroblasts, there is increased sensitivity to the TNF-related apoptosis ligand (TRAIL) that selectively targets cancer cells but not non-malignant normal cells [187].

## 6. Conclusions

From the discovery of Warburg in the 1920s till today of 2021, the Warburg effect has received considerable attention, and the number of publications related to cancer cell metabolism is rapidly increasing. Therefore, to treat malignant tumors, it is important to comprehend the metabolic changes in cancer cells.

Among the substances that cells metabolize during glycolysis (glutamine, FA, and glucose), the Warburg effect is associated with the process that catalyzes glucose. Normal cells go through three steps (glycolysis process, TCA cycle, and electron transport system) in a normoxic environment and generate 38 ATPs per glucose molecule. Under hypoxia, normal cells produce two ATPs per glucose molecule as well as lactic acid solely through the glycolysis process. However, since cancer cells prefer anaerobic glycolysis under both normoxia and hypoxia, they require more glucose to produce the same energy as normal cells. One method being applied to detect tumors is by using the difference in metabolism between normal cells and cancer cells, and metabolic anticancer drugs are also being studied along with cytotoxic chemotherapy, targeted anticancer drugs, and cancer immunotherapy.

In cancer cells, proteins related to glycolysis are either upregulated or downregulated, and these proteins can be stabilized by DUBs or prevented from degradation by E3 ligases. Therefore, if UPS can be applied to control the expression levels of proteins related to glycolysis, it can prevent cancer cells from obtaining energy and, thus, be used as a new anticancer therapy. However, the expression levels and cellular roles of DUBs vary depending on the cell type or their substrates. On the other hand, clinical results are insufficient compared to in vivo or in vitro experiments. Therefore, follow-up clinical studies are required.

## Figures and Tables

**Table 1:** The list of DUBs and cellular substrates involved in the Warburg effect.

DUBs	Substrates	Functions	References
USP7	SIRT7	USP7 decreases the K-63 linked ubiquitination level of SIRT7.	[78]
		USP7 decreases the ubiquitin level of SIRT7 as the glucose concentration increased.	
	G6PC	Deubiquitination of SIRT7 by USP7 inhibits the expression of G6PC, a key regulator of SIRT7-mediated glucose production.	
	FoxO1	USP7 deubiquitinates and regulates FoxO1 occupancy in the promoter of the glucose-generating gene.	[82]
USP19	HIF-1α	Silence of USP19 decreased the protein level of HIF-1α.	[92]
	VEGF	Silence of USP19 decreased the mRNA level of *VEGF*.	
	GLUT1	Silence of USP19 decreased the mRNA level of *GLUT1*.	
USP28	c-Myc	USP28 deubiquitinates c-Myc through interaction with Fbw7.	[97]
USP37	c-Myc	USP37 directly binds to c-Myc and deubiquitinates the c-Myc.	[104]
	GLUT1	Depletion of USP37 leads to decreased expression of *GLUT1* mRNAs required for glucose uptake.	
	LDHA	Depletion of USP37 leads to decreased expression of *LDHA* mRNAs required for lactic acid production.	
USP44	FBP1	Knockdown of USP44 improves glucose utilization and lactic acid production capacity by reducing FBP1.	[114]
OTUB2	c-Myc	OTUB2 increases the expression level of c-Myc.	[119]
	HIF-1α	OTUB2 increases the expression level of HIF-1α.	
	GLUT1	OTUB2 increases the expression level of GLUT1.	
	U2AF2	OTUB2 increases the expression level of U2AF2.	
		OTUB2 regulates the Warburg effect via interaction with U2AF2.	
	HK2	OTUB2 increases the expression level of HK2.	
	PGAM1	OTUB2 increases the expression level of PGAM1.	
	PGK1	OTUB2 increases the expression level of PGK1.	
	mTOR	OTUB2 increases the level of phosphorylation of mTOR.	
	AKT	OTUB2 increases the level of phosphorylation of AKT.	
OTUD6B	HIF-1α	Overexpression of OTUD6B increased the ubiquitination level of HIF-1α and decreased protein level of HIF-1α.	[126,127]
	pVHL	OTUD6B inhibits pVHL from proteasome degradation through binding with elongin B and enhancing the interaction with pVHL-elongin C.	
OTUD7B	HIF-1α	Knockdown of OTUD7B decreases the protein levels of HIF-1α and increases the K11-linked polyubiquitin chain.	[133]

**Table 2:** The list of DUBs and inhibitors involved in the Warburg effect.

DUBs	Inhibitors	References
USP7	FT671	[168]
	FT827	[168]
	GNE-6640	[169]
	GNE-6776	[169]
	HBX 19,818	[170]
	HBX 28,258	[170]
	HBX 41,108	[171]
	P22077	[172]
	P50429	[173]
	P5091	[174,175]
	vif1	[176]
	vif2	[176]
	XL177A	[177]
	XL188	[178]
USP19	unknown	
USP28	AZ1	[179]
	[1,2,3]triazolo[4,5-d]pyrimidine	[180]
USP37	unknown	
USP44	unknown	
OTUB2	unknown	
OTUD6B	unknown	
OTUD7B	unknown	
Broad spectrum DUB inhibitor	Betulinic acid	[181,182,183,184,185]
	PR-619	[186,187]

## Data Availability

Not applicable.
